# A singular thermodynamically consistent temperature at the origin of the anomalous behavior of liquid water

**DOI:** 10.1038/srep00993

**Published:** 2012-12-18

**Authors:** Francesco Mallamace, Carmelo Corsaro, H. Eugene Stanley

**Affiliations:** 1 Dipartimento di Fisica, Università di Messina and CNR-IPCF, I-98166, Messina, Italy; 2 Department of Nuclear Science and Engineering, Massachusetts Institute of Technology, Cambridge MA 02139 USA; 3 Center for Polymer Studies and Department of Physics, Boston University, Boston, MA 02215 USA

## Abstract

The density maximum of water dominates the thermodynamics of the system under ambient conditions, is strongly *P*-dependent, and disappears at a crossover pressure *P*_cross_ ~ 1.8 kbar. We study this variable across a wide area of the *T*–*P* phase diagram. We consider old and new data of both the isothermal compressibility *K_T_*(*T*, *P*) and the coefficient of thermal expansion *α_P_*(*T*, *P*). We observe that *K_T_*(*T*) shows a minimum at T* ~ 315±5 K for all the studied pressures. We find the behavior of *α_P_* to also be surprising: all the *α_P_*(*T*) curves measured at different *P* cross at T*. The experimental data show a “singular and universal expansivity point” at T* ~ 315 K and *α_P_*(T*) ≃ 0.44 10^−3^ *K*^−1^. Unlike other water singularities, we find this temperature to be thermodynamically consistent in the relationship connecting the two response functions.

Water is a ubiquitous substance fundamental to life on earth. It is also a complex liquid with a large number of counterintuitive anomalies. These two facts alone make water a most intriguing topic for research^1^. The best known of water's unusual properties are its density and viscosity at ambient pressure. Below its density maximum at 4°C, water expands and becomes more compressible and less viscous. Other anomalous behaviors include those associated with such thermal response functions as isothermal compressibility *K_T_*, isobaric heat capacity *C_P_*, and thermal expansion coefficient *α_P_*. At ambient pressure, when these response functions are extrapolated from their values in the metastable supercooled phase of water (located between the homogeneous nucleation temperature *T_H_* = 231 K and the melting temperature *T_M_* = 273 K), they appear to diverge at a singular temperature (*T_S_* ≃ 228 K)^1^. Water also becomes glassy below *T_g_* ≈ 130 K and in that region can exist in two distinct amorphous forms (i.e., it is “polymorphous”)^2^. The low-density-amorphous (LDA) and highdensity-amorphous (HDA) phases exist below *T_g_*, and by tuning the pressure the system can be transformed back and forth between the two^2^. Immediately above *T_g_* water becomes a highly viscous fluid and at *T_X_* ≈ 150 K crystallizes. The region between *T_X_* and *T_H_* represents a “No-Man's Land” within which water can be studied only if confined in small cavities so narrow the liquid cannot freeze, or if it is located around macromolecules such as the hydration water around proteins^3^.

Water is thus an exciting research topic, and an enormous number of studies have probed the physical reasons for its unusual properties. A convergence of experimental and theoretical results strongly indicates that the key to understanding water's anomalous behavior is the role played by hydrogen bond (HB) interactions between water molecules. All three principal hypotheses proposed to understand water, i.e., the stability-limit^4^, the singularity-free^5^, and the liquid-liquid critical point (LLCP)^6^ scenarios agree in this regard.

The LLCP approach makes two basic assumptions: (i) as *T* decreases, the HBs cluster and form an open tetrahedrally-coordinated HB network, and (ii) water “polymorphism” exists. If we begin with the stable liquid phase and decrease *T*, the HB lifetime and the cluster stability increase, and this structure continues through the No-Man's Land down to the amorphous phase region where water is polyamorphic. Hence liquid water has local structure fluctuations, some of which are like LDL and others like HDL, with an altered local structure that is a continuation of the LDA and HDA phases^6^. In HDL, which predominates at high *T*, the local tetrahedrally coordinated HB structure is not fully developed, but in LDL a more open, “ice-like” HB network appears. Water anomalies can reflect the “competition” between these two local forms of liquid. The LLCP scenario also predicts a special locus, the Widom line, in the *T*–*P* phase diagram at which the water response functions are at their maximum values^7^. Unfortunately, the study of this line and the associated polymorphic transition in bulk water is hampered because it lies well within the No-Man's Land, but the crystallization inside this region can be retarded by confining water within nanoporous structures so narrow that the liquid cannot freeze^3^, or within its own ice phase^8^, or on a protein surface (hydration water)^9^.

The experiments done on water in nanopores^3^,^10^,^11^,^12^ have shown that, when *T* is lowered, at a certain point the water HB lifetime increases by approximately six orders of magnitude, clearly indicating the presence of LDL and HDL inside the supercooled region^13^ and indicating the location of the Widom line^10^,^12^. At ambient pressure the Widom line is crossed at *T_W_*(*P*) ≃ 225 K where: (i) a fragile-to-strong dynamic crossover occurs^7^,^10^, (ii) the Stokes-Einstein relation is violated^11^,^14^, and (iii) the LDL local structure predominates over the HDL^12^,^14^. These findings on confined water have been confirmed by a number of different experiments^8^,^15^ and MD studies^7^,^14^. As yet there has been no proof that such a reality exists in bulk water, and thus water's anomalous behavior remains an open scientific question. Here we attempt to clarify the situation by taking into account bulk water data of thermodynamical response functions *ρ* and *K_T_*, expansivity *α_P_*, the transport parameter, viscosity *η*, and self-diffusion coefficient *D_S_* as a function of both temperature and pressure. In this way we test, across a wide area of the *T*–*P* phase diagram, the connection between water anomalies and the local molecular order dominated by HB networking.

## Results

**Figure 1:**
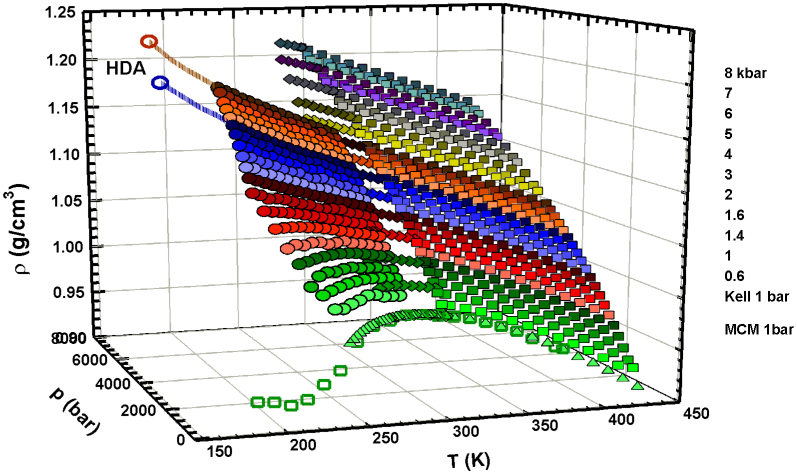
The bulk water density *ρ* as a function of *T* and *P*, in the ranges 150–450 K and 1 bar–8 kbar^16^,^17^,^18^,^19^,^20^,^21^,^22^,^23^,^24^. As it can be observed the density maximum temperature is *P*–dependent and disappears for *P* > 2 kbar. It is also evident that the pressure increase is accompanied by a complete change in the *ρ*(*T*) curvature (from negative to positive) at such a pressure. Are also reported two density values measured in HDA respectively at 3 kbar and 4 kbar (dotted lines evidence a continuity between these HDA values and the bulk water *ρ* data). The open green squares represent the density measured, at 1 bar, in confined water inside the no man's land, where there is also a density minimum located at ~ 200 K.

It is structurally interesting to note (Figure 1) that one of the most important water anomalies, i.e., the density maximum that dominates system thermodynamics under ambient conditions, is strongly *P*-dependent. If we increase *P*, the density maximum moves to a lower *T* (e.g., at *P* = 1 kbar it is *T* ~ 245 K). Figure 1 shows the overall fluctuations of the density *ρ*(*T*, *P*) and clearly indicates this behavior. The reported data^16^,^17^,^18^,^19^,^20^,^21^,^22^,^23^ refer to bulk and emulsified water (with water droplets of size 1–10 *μ*m)^23^. Note that, in addition to being *P*-dependent, the temperature of density maximum disappears when *P* > 1.8 kbar. Note also that at this *P* there is a complete change in the *ρ*(*T*) curvature ((*∂ρ*/*∂T*)*_P_*) from negative to positive. Figure 1 also shows two density values at ~ 155 K measured in HDA at 3 kbar and 4 kbar (the dotted lines indicate the continuity between these HDA values and the bulk water *ρ* data). Note that although these HDA densities measured at very high pressures are of the order of 1.2 g/cm^3^ (or even higher), the value of the LDA density measured at 1 bar and 130 K is ~ 0.94 g/cm^3^, a value that agrees with the values measured in confined water (MCM nano-tubes) inside the No Man's Land, where a density minimum is also seen at *T* ~ 200 K (green open squares shown in Fig. 1^24^).

From this complex *ρ*(*T*, *P*) behavior we note that, because the water density maximum is strongly *P*,*T* dependent and disappears at a certain crossover pressure (*P*_cross_ ~ 1.8 kbar), our understanding of the thermodynamic relevance of the density maximum must be adjusted. Perhaps this crossover pressure and some quantity related to (*∂ρ*/*∂T*)*_P_* has a physical significance we do not yet understand.

**Figure 2:**
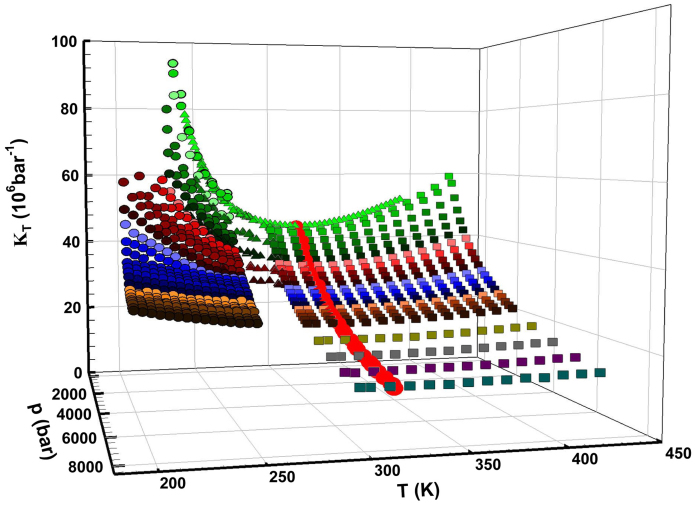
The bulk water isothermal compressibility *K_T_*(*T*, *P*) in the same *P*, *T* ranges of the density data illustrated in the Figure 1, the pressures are identified by means of the same symbols^16^,^18^,^19^,^23^,^25^,^26^,^27^. A simple data inspection shows: i) two distinct behaviors of the *K_T_* dependence, at the different pressures, in the high and low temperature regimes; ii) at all the reported pressures the *K_T_*(*T*) curves present a minimum value located at the same temperature T* ~ 315 K ±5 K; iii) also for *K_T_*, like for *ρ*, it seems that *P*_cross_ is at the borderline of two regions; one of very large volume fluctuations (*P* < *P*_cross_, and *T* < T*) and a second one where 〈*δV*^2^〉 is comparable with that of the liquid in its stable phases.

On this basis we consider the isothermal compressibility *K_T_* (*K_T_* = (*∂* ln *ρ*/*∂* ln *P*)*_T_* = −*V*^−1^(*∂V*/*∂P*)*_T_*) in the same *P* and *T* intervals previously reported for *ρ*(*T*, *P*). Figure 2 shows the literature data of *K_T_*(*T*, *P*)^16^,^18^,^19^,^23^,^25^,^26^,^27^, which, as is well-known, is related to volume fluctuations *δV* as *K_T_* = 〈*δV*^2^〉*_P,T_*/*k_B_TV*. Inspecting the data we see (i) two distinct *K_T_* behaviors in the high and low *T* regimes, (ii) for the pressures in the 1 < *P* < 8 kbar range the corresponding *K_T_*(*T*) curves show a minimum (red dots) that is located at T* ~ 315 ± 5 K, and (iii) as observed for *ρ*, for *K_T_* *P*_cross_ is the borderline between two regions, one with large fluctuations in volume (*P* < *P*_cross_, and *T* < T*) and the other with fluctuations 〈*δV*^2^〉 comparable to those of liquid in its stable phases. Regarding the first and third considerations, Fig. 2 clearly shows that the *P* effect on *K_T_* in the low *P*-*T* regime (including the supercooled phase) is more and more pronounced than that in the high-*T* region (*T* > T*). This is due to the HB network structure (characteristic of the supercooled region and the primary factor behind water's anomalies), which is less dense and more compressible than the HB network at high *T*. This supports the primary assumption of the LLCP model, that the LDL water phase is more pronounced in the low *T* regime and the HDL in the high *T* regime. Figure 2 shows data indicating that the onset of the LDL (i.e., the HB network) occurs near T*.

## Discussion

**Figure 3:**
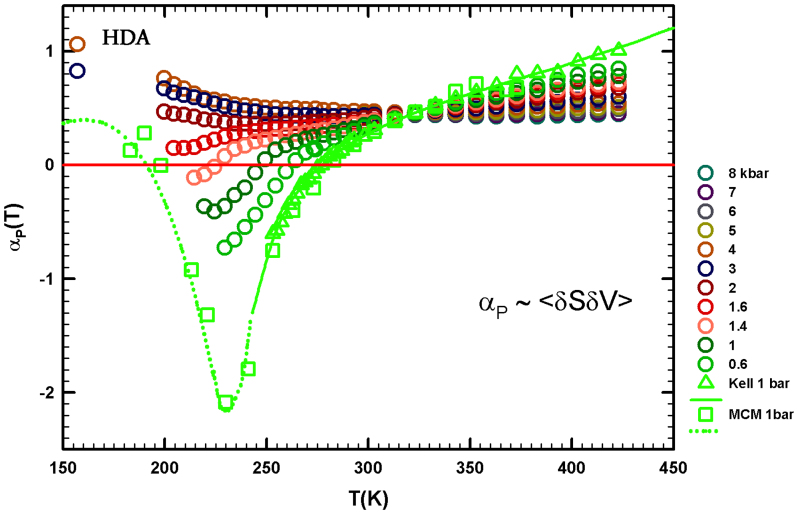
The bulk water coefficient of thermal expansion *α_P_*(*T*) in the same T,P intervals of the previous figures. It is clearly observable that all the *α_P_*(*T*) curves evaluated at a certain pressures cross at the same point: T* ~ 315 K with 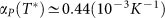.

Figure 1 shows the role of the density derivative as a function of *T*. Hence we consider the coefficient of thermal expansion *α_P_* = −(*∂* ln *ρ*/*∂T*)*_P_* = −*V*^−1^(*∂S*/*∂P*)*_T_*, representing the entropy and volume cross-correlations 〈*δSδV*〉 to be *α_P_* = 〈*δSδV*〉/*k_B_TV*. Regarding this response function, note that, in simple liquids, *δS* and *δV* fluctuations become smaller as *T* decreases and are positively correlated, whereas in water they become more pronounced and, for *T* < 277 K at ambient *P*, are anticorrelated^1^. The local order in water is the microscopic cause of these behaviors. As in compressibilty, the *P*–*T* behavior of *α_P_* is surprising and, as shown by Fig. 3, T* is the border between two different behaviors. In the large *P*-range explored, all the *α_P_*(*T*) curves measured at different pressures cross, within the error bars, at the same temperature T*. Specifically, the experimental data show a “singular and universal expansivity point” at T* ~ 315 K and *α_P_*(T*) ≃ 0.44 10^−3^ *K*^−1^. From these data we can see that, for *T* > T*, the thermodynamic behavior of water is exactly the same as a normal fluid for all the available *P*–*T* values, but that the situation changes in the remaining regions of the phase diagram where, as a function of *P*, different behaviors are observed. For *P* > *P*_cross_ the *δS* and *δV* fluctuations are positively correlated but for *T* < T* they increase as *T* decreases. In *P* = 3 kbar and *P* = 4 kbar there is an apparent continuity between bulk water and its HDA phase. For *P* < *P*_cross_ the *α_P_*(*T*) evolution is more complex, i.e., *α_P_*(*T*) decreases as *T* decreases and, when *P* < 1.6 kbar, anticorrelation processes appear. According to the data, *α_P_*(*T*) decreases up to a certain flex point and, after a further decrease in *T*, goes to a minimum, the value of which decreases as the pressure increases. The exact values and *T*-positions of these minima (for *P* < *P*_cross_) are not clearly defined in the bulk water data for *α_P_*(*T*), but their overall behavior seems fully consistent with a data evolution similar to that observed in confined water. Note that in the case of confined water (MCM-41) such a minimum temperature is coincident with that of the fragile-to-strong dynamical crossover and the Widom line, which at ambient pressure is *T_W_*(*P*) ≃ 225 *K*.

Although these *α_P_*(*T*) minima and their relations with the Widom line do not represent the core of the actual work, the expansion coefficient behavior for *T* > T* is enough to clarify the water properties from a thermodynamical point of view by considering that these data represent the entropy and volume cross-correlation. As mentioned above, two different behaviors are present in 〈*δSδV*〉/*k_B_TV* for pressures above and below *P*_cross_. Note that anticorrelations are possible only for *P* < *P*_cross_, and that the maximum anticorrelation strength occurs at ambient pressure, decreases with increasing *P*, and vanishes at *P*_cross_. This is clearly linked to the HB networking process that characterizes the local order of water: as *T* decreases inside the supercooled regime it affects the growth (with increasing stability) of the molecular water structure and gives rise to a sudden entropy decrease. In contrast, pressure effects cause a progressive decrease in HB clustering. Figure 1 shows the *ρ*(*T*, *P*) behavior. The density maximum characterizing water disappears near *P*_cross_ after which the system behaves as a normal liquid. This is a strong indication that the HB network, i.e., the dynamic water clusters organized in a tetrahedral structure, has a low-density local order. If the presence of this HB network, as far as the behavior proposed by the *α_P_*(*T*) data (Figure 3), is or is not consistent with the LLCP approach does not matter with our study; instead, summarizing all the proposed results, here we stress that the water singular temperature T* has a precise thermodynamical consistence lying in the relationship connecting two of the studied response functions: 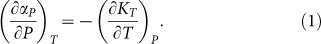
Note that T* represents the liquid bulk water isothermal compressibility minimum temperature and also the crossing point of all the thermal expansion functions in the large phase diagram area, i.e., 200 K < *T* < 430 K and 1 bar < *P* < 8 kbar.

**Figure 4:**
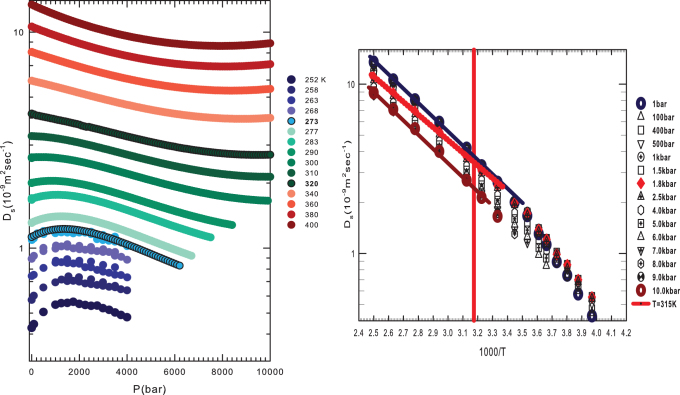
(a) The bulk water self-diffusion coefficient *D_S_* as a function of the pressure in the range 1 bar< *P* < 10 kbar at different temperatures from the supercooled region 252 K to 400 K. (b) The self-Diffusion coefficient *D_S_*, at several different pressures from 1 bar to 10 kbar in an Arrhenius fit (log *D_S_* vs 1000/*T*). The red line represents the T* temperature crossover that identifies two different scenarios, Arrhenius for *T* > T* and super-Arrhenius for *T* < T*. I.e. the transition of water from a simple to a strong interacting structured liquid.

We now examine self-diffusion coefficient *D_S_*(*T*, *P*) data. This is a dynamic quantity from which we can determine further information about T*. Figure 4(a) shows *D_S_* measured in bulk water as a function of the pressure (1 bar < *P* < 10 kbar) at several temperatures in the range 252 K–400 K. The *D_S_*(*T*, *P*) data in the interval 252 K < *T* < 290 K are measured using Nuclear Magnetic Resonance (NMR)^28^. The data for *T* > 300 K assume the validity of the Stokes-Einstein relation and are derived from viscosity data available in the literature^29^. Note that, in the dynamics of the system, T* ~ 315 K marks the crossover between two different physical realities: below T*, the self-diffusion coefficient has a maximum that for *T* = 252 K is located at ≈ 1600 bar and that, as *T* increases, evolves at the lowest *P* and disappears near T*. When *T* > T*, the *D_S_*(*P*) behavior is more regular. Figure 4 (b) shows these data at a given pressure in an Arrhenius plot (ln *D_S_* vs. 1/*T*), and they further clarify the properties of water. Note that when 1 bar < *P* < 10 kbar, T* (vertical red line) marks two different regions: for *T* > T* the thermal behavior of the self-diffusion coefficient is simply Arrhenius (*D_S_* = *A* exp(*E*/*k_B_T*)), but in the temperature range from T* to the supercooled region (the lowest *T* is 252 K) the behavior is super-Arrhenius. Hence T* marks a transition from an high-*T* region characterized by a water molecular dynamics with only one energy scale (the Arrhenius energy) to another typical of supercooled glass-forming liquid systems in which the temperature decrease gives rise to increasing intermolecular interactions (correlations in the time and length scale, i.e., dynamic clustering). In the water case this is the onset of the HB tetrahedral network. As in complex liquids, the interaction process originates in the disordered and finite correlation regions (finite polydisperse dynamic clustering) reflected in the transport parameters (relaxation times, viscosity, and self-diffusion) by means of a super-Arrhenius behavior or a multi-relaxation in the time evolution of the density-density correlation functions. Liquid state theory suggests the presence of an onset temperature marking a crossover from normal liquid behavior to supercooled liquid behavior^30^,^31^,^32^,^33^,^34^. Above that the transport is Arrhenius and below that correlations cause activation barriers to grow with a growing scale resulting in super-Arrhenius behavior^30^,^33^,^34^,^35^. Finally, Figure 4(b) shows the Arrhenius activation energy (*T* > T*) obtained as *E* = 15.2 ± 0.5 kJ/mol, i.e., the HB energy value, fully supporting the primary role of HBs in the properties of water.

Such a picture, derived from transport data, represents the dynamic aspect of the important reality that also characterizes the thermodynamic response functions in bulk water (Figs. 1–3). However the importance of the T* in water can be fully evaluated only by considering in an unitary way all the studied quantities. From the structural point of view, T* may be the onset temperature of the HB clustering, the magic point at which liquid water becomes a complex material. In addition, the experimental data, the large *P-T* phase diagram, and the thermodynamic consistency shown in Eq. (1), all indicate that T* plays a primary role in the physics of water physics and is the source of its anomalies.

## Author Contributions

All authors contributed extensively to the work presented in this paper.
